# Effects of remimazolam and propofol on parasympathetic activity during general anesthesia induction in patients with severe aortic stenosis: a randomized controlled trial utilizing high-frequency variability index

**DOI:** 10.1007/s10877-026-01433-z

**Published:** 2026-04-02

**Authors:** Taichi Kotani, Mitsuru Ida, Yusuke Naito, Nobuhiro Tanaka, Masahiko Kawaguchi

**Affiliations:** Department of Anesthesiology, Nara Medical University, 840 Shijo-cho, Kashihara, 634-8522 Nara Japan

**Keywords:** Remimazolam, Propofol, Autonomic Nervous System, Parasympathetic Nervous System, Aortic Valve Stenosis, High-frequency Variability Index

## Abstract

In this study, we aimed to compare the effects of remimazolam and propofol on parasympathetic activity during general anesthesia induction in patients with severe aortic stenosis using heart rate variability (HRV) analysis. In this single-center randomized controlled trial, 28 patients scheduled for elective transcatheter aortic valve replacement were assigned to receive either remimazolam or propofol for anesthesia induction at a tertiary emergency medical facility. Parasympathetic activity was assessed using the high-frequency variability index (HFVI), derived from spectral analysis of HRV based on electrocardiographic R–R intervals. HFVI was recorded for 3 min before and after induction. Remimazolam or propofol was administered at 6 mg/kg/h or 2.0 µg/ml via target-controlled infusion, respectively. The primary outcome was the difference in mean HFVI values recorded between the 3-min period before and after induction. Hemodynamic parameters, including mean blood pressure, heart rate, cardiac output, stroke volume variation, pulse pressure variation, and dynamic arterial elastance, were also measured. Baseline HFVI values did not differ significantly between groups. After induction, HFVI decreased significantly more in the remimazolam group than in the propofol group (ΔHFVI: 16 vs. 3, *P* = 0.010). Heart rate increased in the remimazolam group but decreased in the propofol group (*P* = 0.006). No significant intergroup differences were observed in other hemodynamic parameters. These findings suggest that remimazolam may be associated with distinct autonomic responses during anesthesia induction.

## Introduction

 Propofol is commonly used for general anesthesia, but causes vasodilation and reduced cardiac contractility, which can result in hypotension [1, 2]. Preventing hypotension during anesthesia induction is essential because intraoperative hypotension has been linked to myocardial injury, acute kidney injury, and perioperative mortality [3]. Patients with severe aortic valve stenosis are particularly vulnerable to hemodynamic instability during induction and therefore require especially careful management [4].

Remimazolam is a short-acting intravenous benzodiazepine anesthetic [5]. Early Japanese trials reported that remimazolam has fewer cardiodepressant effects than propofol, suggesting its potential for safer use in patients with cardiovascular comorbidities [6–8]. Although these trials excluded patients undergoing cardiovascular surgery, more recent studies have shown that remimazolam is effective in that setting [8]. A randomized controlled trial also reported that remimazolam was associated with a lower incidence of hypotension during anesthesia induction than propofol in patients with severe aortic valve stenosis [9]. However, the mechanisms underlying this difference remain unclear. One study on healthy individuals found that the sympathetic nervous system becomes more dominant during anesthesia induction with propofol than with remimazolam, possibly as a compensatory response to hypotension [10]. In contrast, patients with aortic valve stenosis are prone to hypotension during anesthetic induction, prompting a shift in contemporary practice from treating hypotension to preventing it. Moreover, those with left ventricular hypertrophy or heart failure, including patients with aortic valve stenosis, exhibit chronic activation of the sympathetic nervous system [11]. Consequently, in these patients, autonomic responses to anesthetics may differ from those in healthy individuals; however, these differences remain inadequately investigated.

To date, no studies have examined the effects of remimazolam on autonomic function during anesthesia induction in patients with advanced cardiac pathology; prior work comparing remimazolam and propofol using HRV during induction has largely focused on relatively healthy populations [10]. To investigate peri-induction autonomic patterns in a feasible bedside manner, we selected the high-frequency variability index (HFVI) as the primary endpoint. HFVI is a commercially available HRV-derived index that reflects relative parasympathetic modulation based on the high-frequency component and is conceptually aligned with the analgesia-nociception index [12]. Unlike conventional spectral HRV analyses, which often require offline processing, HFVI provides real-time numeric output and is available for routine clinical use in our nation, Japan [12, 13]. Recent clinical reports have treated HFVI as a continuous measure and suggest that the magnitude of change (ΔHFVI), rather than a single threshold, can be clinically interpretable—showing minimal, non-meaningful shifts in low-impact settings but larger changes in clinically meaningful physiological transitions [13–16]. Because severe aortic stenosis is characterized by autonomic dysfunction and limited compensatory reserve, a clinically deployable parasympathetic index may help characterize induction-related autonomic vulnerability and agent-specific autonomic patterns [11, 17]. We hypothesized that remimazolam and propofol would differ in autonomic compensation, with particular emphasis on parasympathetic modulation in these patients. Therefore, we prespecified the peri-induction change in HFVI (ΔHFVI) as the primary outcome and designed a randomized controlled trial to compare the effects of remimazolam and propofol on autonomic nervous system activity during anesthesia induction in patients with severe aortic valve stenosis.

## Methods

### Study design

This single-center, single-blind, randomized controlled trial received ethical approval from the local ethics committee (approval number: 3715, Chairperson: Prof. Kazuhiko Tsuruya) on March 8, 2024. The study was registered at the Japan Registry of Clinical Trials (jRCT1052230204). All patients provided written informed consent. The study followed the Consolidated Standards of Reporting Trials (CONSORT) guidelines [18]. Trial registration: jRCT1052230204. (URL, https://jrct.mhlw.go.jp/latest-detail/jRCT1052230204).

### Study population

Patients aged ≥ 20 years scheduled to undergo elective transcatheter aortic valve replacement (TAVR) under general anesthesia were eligible. Exclusion criteria included lack of informed consent and hypersensitivity to the study medication. Patients with chronic atrial fibrillation, pacemaker implantation, or diabetes mellitus with neuropathy—conditions that interfere with critical care monitoring—were also excluded. Eligible patients were recruited after routine preoperative assessments at our institution. Following written informed consent, 28 patients were enrolled between April 1, 2024, and May 23, 2025.

### Randomization

Patients were randomly assigned in a 1:1 ratio (remimazolam vs. propofol) using a computer-generated randomization table created by a co-investigator (Y.N.) who had no role in patient management.

### Assessment of heart rate variability

Since 2022, the high-frequency variability index (HFVI; Mdoloris Medical Systems, Loos, France), a numerical indicator of parasympathetic tone, has been commercially available in Japan. Using this monitor, we attempted to analyze the effects on the autonomic nervous system, particularly the parasympathetic nervous system. We assessed parasympathetic activity using the HFVI during anesthesia induction. HFVI analysis involves attaching a dedicated monitor to the chest and measuring heart rate variability (HRV) from electrocardiographic R–R intervals. Spectral analysis separates HRV into high-frequency (HF) and low-frequency (LF) components. The LF component, with a peak in the 0.04–0.15 Hz range, reflects both sympathetic and parasympathetic activity, whereas the HF component, with a peak in the 0.15–0.4 Hz range, reflects parasympathetic activity alone. HFVI is a reconstituted waveform derived solely from the HF component, providing a measure of relative parasympathetic activity [13]. HFVI values range from 0 (minimum) to 100 (maximum parasympathetic activity).

### General anesthesia induction

Routine oral medications were continued perioperatively, except for angiotensin receptor blockers and angiotensin-converting enzyme inhibitors, which were withheld. Oral β-blockers were continued. Patients did not receive preoperative medication on the day of surgery. Before the patient entered the operating room, we synchronized the time on all measuring instruments to the second to align the recorded values. In the operating room, patients were monitored in the supine position. An arterial catheter was placed and connected to a FloTrac sensor (Edwards Lifesciences, Irvine, CA, USA) to record hemodynamic parameters every 20 s. An HFVI sensor (HFVI V1 Plus, Mdoloris Medical Systems) was attached to the anterior chest, and HFVI values were displayed on the Root^®^ monitor (Masimo) through the HFVI MOC-9 module. The monitor generated average HFVI values over 120-s (HFVIi) and 240-s (HFVIm) intervals. After loss of consciousness, anesthetic depth was titrated to achieve a patient sedation index (PSI) of 25–50 using a SedLine^®^ monitor (Masimo, Irvine, CA, USA). Left and right spectral edge frequency (SEF) values were also recorded. Data were acquired using the Masimo Instrument Configuration Tool (MICT; Masimo), which automatically exports HFVI, PSI, and associated parameters from the Root monitor.

**Fig. 1:**
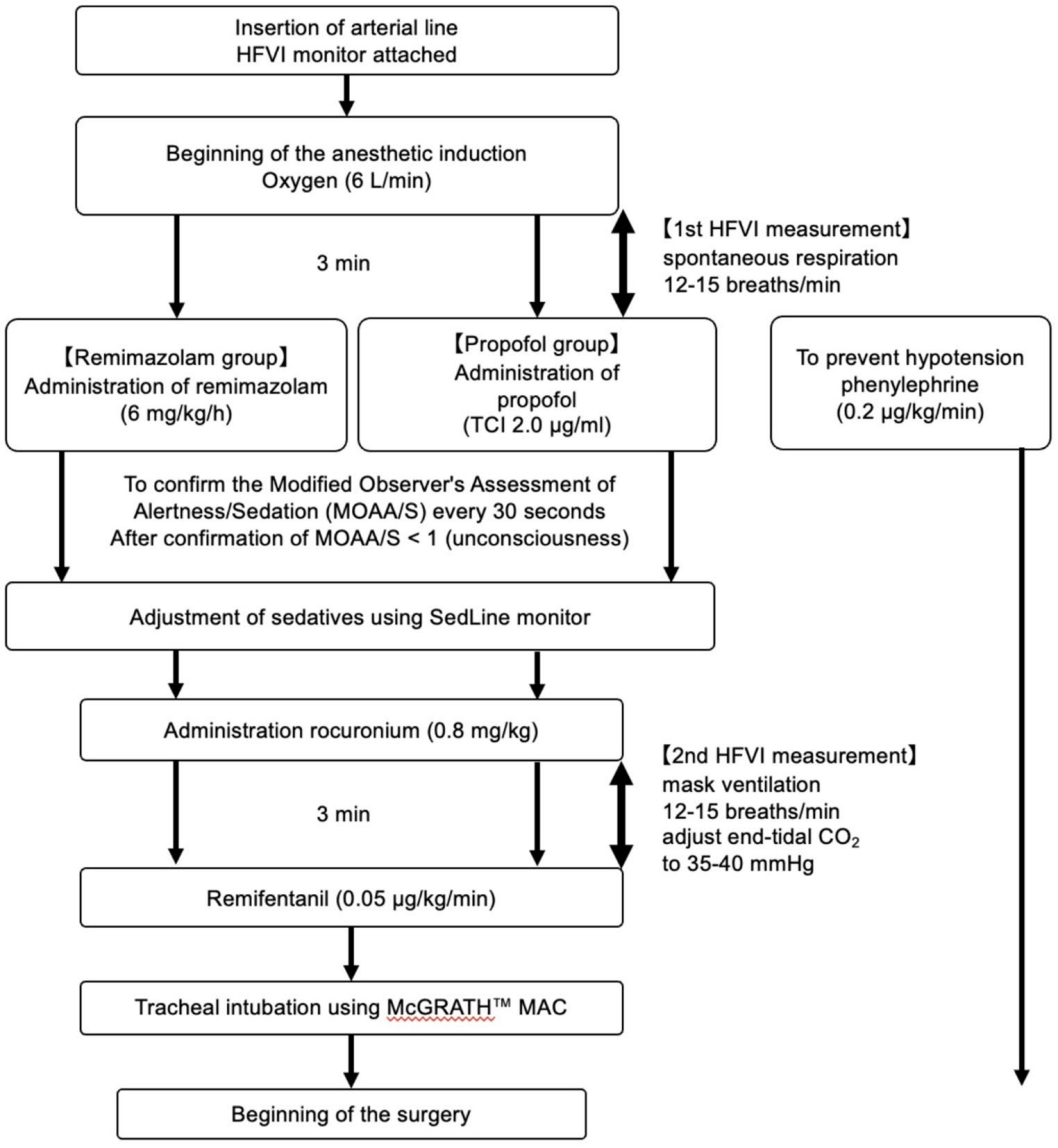
Schema of drug adjustment and anesthetic procedures

A schematic of drug adjustment and anesthetic procedures is shown in Fig. 1. Anesthesia was administered by a separate board-certified anesthesiologist, ensuring that both investigators and patients were blinded to group allocation. As the patient was under anesthesia, blinding was maintained at the patient level, but anesthesia providers could not be blinded.

The first HFVI measurement (1st HFVI) was performed for 3 min at the start of 6 L/min 100% oxygen administration. Respiratory rate was maintained at 12–15 breaths/min using a metronome to minimize respiratory modulation of HFVI [19, 20]. The remimazolam group (*n* = 14) received remimazolam at 6 mg/kg/h for induction. The induction rate of 6 mg/kg/h was selected based on prior clinical experience in elderly patients with severe aortic stenosis reported by Nakanishi et al. [21]. The propofol group (*n* = 14) received propofol at an effect-site concentration of 2.0 µg/ml via target-controlled infusion (Diprifusor^®^ model, TE-SS830T, Terumo, Tokyo, Japan). An attending anesthesiologist assessed the Modified Observer’s Assessment of Alertness/Sedation (MOAA/S) every 30 s [22]. After MOAA/S < 1 (unconsciousness) was confirmed, the remimazolam infusion rate was adjusted stepwise according to PSI (decrease by 0.1 mg/kg/h if PSI ≤ 25 for > 30 s; increase by 0.2 mg/kg/h if PSI ≥ 50 for > 30 s). Under this PSI-targeted titration, infusion rates during the post–loss-of-consciousness period were typically 0.6–1.0 mg/kg/h, as adopted from our previous TAVR anesthesia protocol [9]. The propofol target concentration was adjusted within a prespecified range of 1.8–2.2 µg/ml. Both anesthetics were titrated using a predefined PSI-guided protocol (target PSI 25–50, decrease by 0.1 µg/ml if PSI ≤ 25 for > 30 s; increase by 0.2 µg/ml if PSI ≥ 50 for > 30 s). After loss of consciousness, rocuronium (0.8 mg/kg) was administered to facilitate tracheal intubation. The second HFVI measurement (2nd HFVI) was obtained for 3 min during mask ventilation after rocuronium administration. Ventilation was maintained at 12–15 breaths/min and end-tidal CO_2_ at 35–40 mmHg. During both HFVI measurement periods, mean blood pressure (mBP), heart rate (HR), cardiac output (CO), stroke volume variation (SVV), pulse pressure variation (PPV), and dynamic arterial elastance (Ea_dyn_ = PPV/SVV) were recorded every 20 s, while HFVIi, HFVIm, and PSI were recorded every 2 s.

As patients with severe aortic valve stenosis are prone to hypotension during anesthesia induction [9], fluid therapy was initiated with 1% glucose-containing acetate Ringer’s solution at 5 ml/kg/h, along with continuous vasopressor infusion at the onset of anesthetic administration. Phenylephrine (0.2 µg/kg/min), a pure α-adrenergic agonist, was selected for preventing hypotension because β-adrenergic agonists, such as ephedrine and norepinephrine, were avoided due to their known effects on HFVI [12, 23, 24]. Remifentanil was initiated after completion of the 2nd HFVI measurement.

In this trial registry, an additional secondary outcome was prespecified as the change in HFVI during rapid pacing at the time of TAVI valve insertion, including peri-balloon aortic valvuloplasty (BAV) phases. We attempted to collect these data; however, during rapid pacing and the peri-BAV phases, temporary apnea (cessation of ventilation) was repeatedly required for procedural reasons. Because HFVI analysis requires a stable respiratory condition and a minimum respiratory rate (9 breaths/min) for valid spectral analysis, these measurements were not considered analyzable and were therefore excluded from the final outcome analysis [13].

### Study outcomes

The primary outcome was the change in HFVI (ΔHFVI), defined as the difference between the 3-min means of the 1 st and 2nd HFVI measurements. We calculated both ΔHFVIi and ΔHFVIm. The primary endpoint was ΔHFVIi, while secondary endpoints included ΔHFVIm and changes in mBP, HR, CO, and Ea_dyn_ between the two measurement periods.

### Sample size calculation

Sample size estimation was based on clinical data from routine use of HFVI during anesthesia induction for TAVR. In our clinical practice, under the same conditions as the present study protocol, the mean change in HFVIi (ΔHFVIi) was 10 in the remimazolam group and 1 in the propofol group. The mean ΔHFVI difference between groups was 9.0, with a pooled standard deviation of 7.5. Power analysis indicated that 11 patients per group were needed to achieve 80% power (1 − β = 0.80; β = 0.20) at α = 0.05. Accounting for 20% attrition, our enrollment target was 14 patients per group.(Table 1)

**Table 1:** Demographic data of the two groups

	Remimazolam (*n* = 14)	Propofol (*n* = 14)
Age (years)	81.6 (5.7)	82.2 (2.9)
Sex (F/M)	12/2	9/5
Height (cm)	151.4 (8.5)	149.6 (9.0)
Weight (kg)	53.8 (10.0)	51.8 (9.3)
EuroSCORE2	6.5 (2.6)	6.1 (1.9)
NYHA (Ⅰ or Ⅱ/Ⅲ or Ⅳ)	11/3	10/4
Clinical frailty scale	3.8 (1.2)	3.6 (0.8)
Echocardiography		
Ejection fraction (%)	64.8 (11.9)	65.8 (10.1)
Peak aortic flow velocity (m/s)	4.3 (0.5)	4.5 (0.5)

Mean (standard deviation) or number. EuroSCORE2, European System for Cardiac Operative Risk Evaluation. NYHA, New York Heart Association functional classification

### Statistical analysis

Continuous data were presented as means (standard deviations), and categorical data as numbers (%). Between-group comparisons were performed using the unpaired t-test for continuous variables and Fisher’s exact test for categorical variables. Normality of continuous variables was assessed using the Shapiro–Wilk test prior to between-group comparisons. In particular, all HFVI-related variables presented in Table 2 (1st HFVIi, 2nd HFVIi, ΔHFVIi; 1 st HFVIm, 2nd HFVIm, ΔHFVIm) were tested within each group, and none showed a significant deviation from normality (all *p* > 0.05). Accordingly, between-group comparisons for these variables were performed using the unpaired t-test. Analyses were conducted with SPSS version 25.0 (IBM Corp., Armonk, NY, USA), and statistical significance was set at *p* < 0.05.

**Table 2:** HFVI during anesthesia induction

	Remimazolam	Propofol	*P* value
1st HFVIi	64.5 (14.2)	59.1 (8.5)	0.232
2nd HFVIi	48.2 (12.2)	56.3 (12.9)	0.099
ΔHFVIi	16.2 (14.5)	2.73 (13.0)	0.010
1 st HFVIm	65.2 (12.1)	60.3 (9.2)	0.237
2nd HFVIm	52.6 (10.4)	57.5 (12.0)	0.265
ΔHFVIm	12.6 (11.5)	2.80 (12.7)	0.042

Mean (standard deviation)

HFVI high-frequency variability index, calculated over 120-s and 240-s intervals, denoted as HFVIi and HFVIm, respectively

1 st refers to the value measured for 3 min before administration of anesthetics, and 2nd refers to the value measured for 3 min after loss of consciousness. Delta(Δ) refers to the difference between the 3-min mean of the 1 st and the 2nd

## Results

**Fig. 2:**
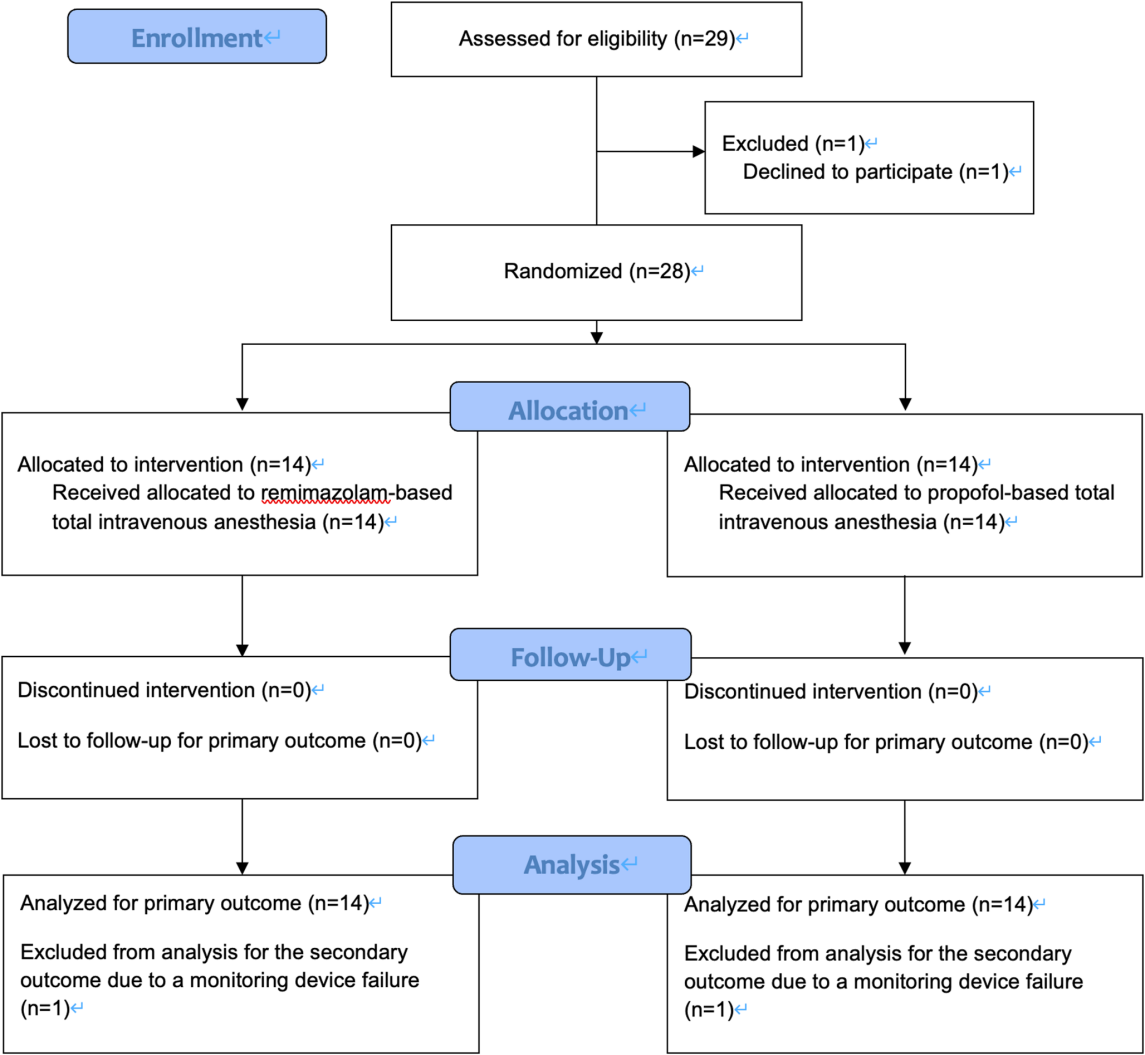
CONSORT 2025 Flow Diagram

The first participant was enrolled on April 5, 2024. A total of 28 patients provided informed consent and were randomly assigned to receive either remimazolam or propofol. Baseline demographic characteristics are summarized in Table 2. One patient in each group was excluded from the analysis of secondary outcomes due to a monitoring device malfunction (Fig. 2). β-blocker use comprised three patients in the remimazolam group (two on carvedilol 10 mg and one on bisoprolol 0.625 mg) and one patient in the propofol group (bisoprolol 0.625 mg); in both groups, β-blockers were continued throughout the perioperative period.

### Primary Outcome

**Fig. 3:**
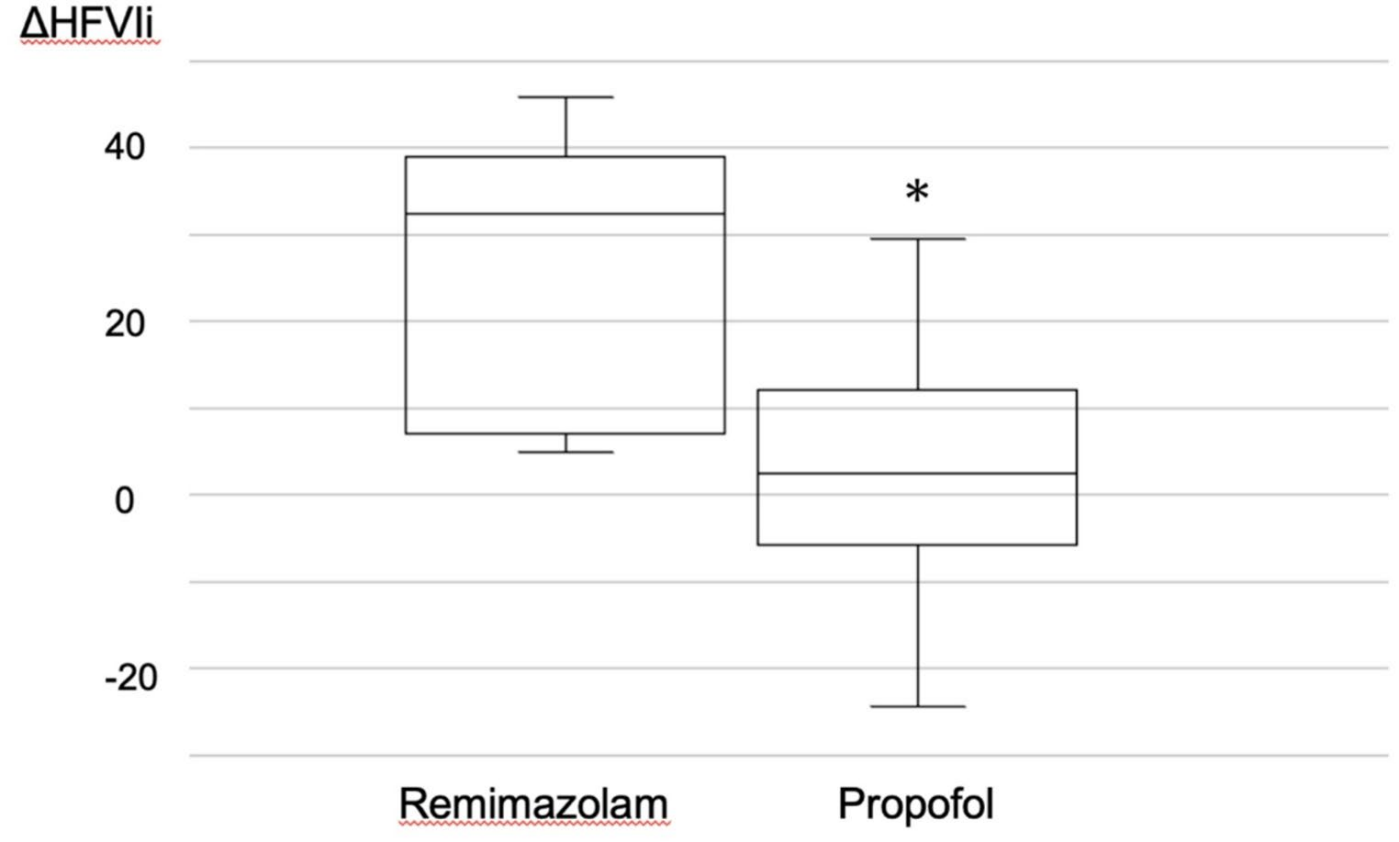
Box-and-whisker plots of ΔHFVIi. Delta(Δ) refers to the difference between the 3-min mean of the 1 st and the 2nd HFVIi, such that a positive value indicates a decrease after induction. ༊P value = 0.01 versus the Remimazolam group

In the remimazolam group, HFVIi decreased from 64 to 48, whereas in the propofol group, it decreased from 59 to 56. The mean changes in HFVIi (ΔHFVIi) were 16 and 3, respectively. The between-group difference in ΔHFVIi was statistically significant (*P* = 0.010) (Table 1, Fig. 3).

### Secondary Outcomes

As shown in Table 2, a significant difference was also observed in the change in HFVIm (ΔHFVIm) between the two groups (*P* = 0.042). With respect to hemodynamic parameters (Table 3), the percentage change in HR was + 9.0% in the remimazolam group and − 6.2% in the propofol group, representing a significant between-group difference (*P* = 0.006). In contrast, no significant differences were observed in the percentage change in mBP. Similarly, SVV, PPV, and Ea_dyn_ values during the 2nd HFVI measurement did not differ significantly between groups (Table 3). Similarly, no significant differences were observed in time to loss of consciousness, PSI, and SEF values between the two groups (Table 4).

**Table 3:** Hemodynamics during anesthesia

	Remimazolam (*n* = 13)	Propofol (*n* = 13)	*P* value
mBP during 1st HFVI (mmHg)	98.2 (13.9)	103.6 (17.0)	0.387
mBP during 2nd HFVI (mmHg)	84.5 (19.6)	89.2 (17.0)	0.510
Percentage change in mBP	−12.7 (20.3)	−12.0 (24.5)	0.939
HR during 1 st HFVI (/min)	67.8 (12.1)	75.0 (13.6)	0.167
HR during 2nd HFVI (/min)	71.4 (12.4)	69.3 (8.1)	0.593
Percentage change in HR	9.0 (15.9)	−6.2 (9.3)	0.006
PPV during 2nd HFVI (%)	10.9 (4.5)	9.6 (3.0)	0.394
SVV during 2nd HFVI (%)	9.8 (4.2)	8.6 (2.8)	0.359
Ea_dyn_ during 2nd HFVI (mmHg/mL)	1.1 (0.2)	1.1 (0.1)	0.845

Mean (standard deviation)

Ea_dyn_: dynamic arterial elastance, defined as the PPV to SVV ratio (PPV/SVV), HFVI: high-frequency variability index, HR: heart rate, mBP: mean blood pressure, PPV: pulse pressure variation, SVV: stroke volume variation

1 st refers to the value measured for 3 min before administration of anesthetics, and 2nd refers to the value measured for 3 min after loss of consciousness

**Table 4:** PSI and SEF values during anesthesia

	Remimazolam	Propofol	*P* value
Time to loss of consciousness (s)	188.5 (87.6)	233.5 (101.3)	0.220
PSI during 1 st HFVI	90.8 (2.1)	90.4 (4.0)	0.783
PSI during 2nd HFVI	51.6 (15)	51.3 (15)	0.970
SEF Left during 2nd HFVI (Hz)	13.5 (4.4)	11.8 (4.1)	0.312
SEF Right during 2nd HFVI (Hz)	13.8 (4.1)	12.4 (3.7)	0.413

Mean (standard deviation)

HFVI: high-frequency variability index, PSI: patient state index, SEF: spectral edge frequency

1 st refers to the value measured for 3 min before administration of anesthetics, and 2nd refers to the value measured for 3 min after loss of consciousness

No deaths occurred within 30 days after the procedure in either group.

## Discussion

This study demonstrated that the effect of remimazolam on parasympathetic activity during general anesthesia induction in patients with severe aortic valve stenosis differed from that of propofol. Specifically, HFVI decreased more in the remimazolam group than in the propofol group, indicating that remimazolam-based induction was associated with lower parasympathetic activity. In this trial, the observed between-group difference in ΔHFVI is better interpreted as a mechanistic signal of differential autonomic modulation during anesthetic induction. As the hemodynamics during general anesthesia induction did not differ between groups under standardized vasopressor support, the present data do not establish a hemodynamic superiority of one agent over the other.

Although HFVI is commonly applied dichotomously with a threshold of 50 for nociception monitoring, several studies have instead treated HFVI as a continuous measure and demonstrated the clinical interpretability of absolute values and changes (ΔHFVI). For example, two observational studies describe minimal, clinically non-meaningful shifts around peripheral nerve block procedures (ΔHFVI ≈ 1–2, with no clinical effect) [13, 14]. In contrast, marked changes have been documented at clinically meaningful shifts around peripheral nerve block procedures (ΔHFVI ≈ 10, with a clinical effect) [15] and at aortic unclamping (ΔHFVI ≈ 13, statistically significant) [16]. In light of this evidence, the ΔHFVIi of 16 observed in the remimazolam group in our study represents a clinically meaningful reduction.

In our previous randomized trial in the same clinical context [9], remimazolam induction was also associated with an increase in heart rate, yet there was no between-group difference in 30-day mortality. This safety signal informed the design of the present mechanistic study. Consistent with that prior experience, no deaths occurred within 30 days in either group in the current trial. Nevertheless, the present study was not powered to detect differences in clinical outcomes, and the heart rate findings should be interpreted in the context of a mechanistic comparison under protocolized hemodynamic management.

The absence of an intergroup difference in mBP should be interpreted in the context of standardized prophylactic phenylephrine infusion. Importantly, the hemodynamic response to phenylephrine is condition-dependent: in patients who are relatively hemodynamically stable, additional α1-mediated vasoconstriction may produce only limited further increases in arterial pressure and can be offset by afterload-related reductions in stroke volume/cardiac output [25]. Therefore, in patients whose blood pressure was already relatively well maintained, as observed in the remimazolam group, excessive increases in blood pressure may have been less likely [26].(Fig. 3)

β-blocker therapy may influence autonomic indices derived from heart rate variability and could therefore act as a potential confounder of HFVI measurements. Several HFVI/HRV studies have excluded patients receiving β-blockers, and catecholaminergic agents have been reported to interfere with HRV-based indices [13, 27]. Nevertheless, available evidence from related autonomic monitoring indices suggests that the impact of β-adrenergic blockade may be modest in some settings (e.g., limited effects of esmolol on ANI and preserved reactivity of the Nociceptive-Level index under chronic β-blockade) [28, 29]. Because β-blocker therapy is standard in elderly patients with severe aortic stenosis and excluding these patients would substantially reduce generalizability, we continued β-blockers per routine care. However, given the small sample size, we cannot fully exclude residual confounding by β-blocker use, and this should be considered when interpreting the observed changes in HFVI.

To date, no research has examined how remimazolam affects the autonomic nervous system during anesthesia induction in patients with severe cardiac conditions such as aortic valve stenosis. A previous study has reported that remimazolam induction is associated with a lower incidence of hypotension than propofol induction in this patient population [9]. That finding suggested that autonomic modulation may underlie the reduced likelihood of hypotension. In the present study, HRV measurements were safely performed in both groups without life-threatening hypotension, which was managed using phenylephrine, a vasopressor with minimal influence on HFVI values [12, 30].

Previous studies have investigated autonomic nervous system responses to anesthetic agents during induction [10, 31, 32]. A randomized controlled trial in participants with American Society of Anesthesiologists (ASA) physical status 1 or 2 found that remimazolam maintained a balanced sympathetic-parasympathetic activity, whereas propofol induced relative sympathetic predominance [10]. The authors proposed that relative sympathetic predominance in the propofol group represented a compensatory response to vasodilation-induced hypotension. Similarly, a retrospective study reported that remimazolam did not alter autonomic activity during induction; however, this finding applied only to healthy individuals (ASA 1 or 2) [33].

The present study targeted patients with severe aortic valve stenosis, under the hypothesis that anesthetic agents may exert different effects on the autonomic nervous system in patients with advanced cardiac pathology, compared with healthy individuals. Aortic valve stenosis is associated with autonomic dysfunction [11]. Chronic left ventricular pressure overload leads to left ventricular hypertrophy and increased wall stress. In this setting, activation of the sympathetic nervous system occurs due to the release of norepinephrine and other neurohumoral factors from the myocardium [34, 35]. Chronic pressure overload also reduces baroreflex sensitivity, thereby diminishing vagal activity and attenuating parasympathetic tone [17, 36]. Consequently, patients with aortic valve stenosis typically demonstrate sympathetic predominance with reduced parasympathetic activity [37, 38].

In such patients, remimazolam may further inhibit parasympathetic activity, thereby maintaining blood pressure during induction [39, 40]. In line with this mechanism, in the present study, heart rates remained stable in the remimazolam group, supporting the notion of parasympathetic inhibition [9]. By contrast, parasympathetic activity showed minimal change during propofol induction. In healthy individuals, propofol may elicit relative sympathetic predominance, thereby sustaining blood pressure. However, in this study, we could not directly assess sympathetic activity because HFVI, which reflects parasympathetic tone by analyzing the HF component of HRV, does not capture the LF component [12]. Given that patients with aortic stenosis already have elevated baseline sympathetic activity, any additional sympathetic activation induced by propofol may have been blunted, resulting in relatively less impact on parasympathetic tone compared with remimazolam.

Concomitant heart failure, which is common in patients with severe aortic stenosis, may influence baseline autonomic function and HRV-derived indices. Frailty and heart failure have been associated with impaired autonomic regulation, including reduced parasympathetic tone and diminished HRV, and reduced HRV is a prognostic marker for adverse outcomes in patients with heart failure or ischemic heart disease [41–44]. In our cohort, patients with advanced symptoms (NYHA class III or IV) were present in both groups and were similarly distributed, supporting that the heart failure symptom burden was broadly balanced between groups by randomization. Nevertheless, NYHA class is an imperfect surrogate for heart failure severity and does not fully capture the severity of autonomic dysfunction. Therefore, residual confounding by underlying heart failure status cannot be completely excluded, and heart failure-related autonomic impairment may have affected absolute HFVI values and/or the magnitude of peri-induction changes observed in this study.

Although our hypothesis focused on differences in autonomic compensatory responses, the HFVI-based methodology was able to quantify parasympathetic activity, but not sympathetic activity. Sympathetic and parasympathetic responses are not invariably reciprocal; however, multiple reports indicate that attenuation of parasympathetic activity is typically accompanied by sympathetic activation [45–47]. In this study, the significant ΔHFVIi observed in the remimazolam group may therefore be interpreted as a concomitant increase in relative sympathetic predominance. Alternatively, this pattern may reflect a compensatory response to hypotension in the remimazolam group, with secondary withdrawal of parasympathetic activity. Importantly, the putative sympathetic activation observed with remimazolam (and not with propofol) may constitute supportive evidence for the hemodynamic stability of remimazolam during induction in patients with severe aortic stenosis.

The present findings should be interpreted primarily as mechanistic rather than as evidence of improved clinical outcomes. However, our data suggest that remimazolam and propofol may be associated with distinct peri-induction autonomic patterns in patients with severe aortic stenosis, even when arterial pressure is maintained under standardized hemodynamic management. Because HFVI is a bedside-available, HRV-derived parasympathetic index in Japan, it may have potential utility for characterizing autonomic vulnerability and agent-specific autonomic responses during induction in high-risk cardiac patients. Severe aortic stenosis and concomitant heart failure are associated with autonomic dysfunction and reduced parasympathetic modulation, which may limit compensatory reserve during induction [11, 17]. Importantly, autonomic dysfunction in severe aortic stenosis has been reported to improve after valve intervention, with recovery of HRV/autonomic balance documented after SAVR or TAVR [37, 48, 49]. In this context, future studies incorporating more granular heart failure and frailty phenotyping, as well as longitudinal assessment of autonomic recovery after TAVR/SAVR, may clarify whether peri-induction HFVI patterns relate to symptom burden, recovery trajectories, or perioperative risk in patients who undergo repeat procedures under general anesthesia.

This study has some limitations. First, generalizability is limited because the trial was conducted at a single center and included only patients undergoing elective TAVR. Second, the initial infusion rates of both agents were based on our routine clinical practice; alternative dosing regimens might have yielded different results. While we cannot demonstrate that anesthetic depth was perfectly equivalent between the two groups, the absence of significant differences in time to loss of consciousness (MOAA/S < 1), PSI, and SEF values supports the validity of this study as a general anesthesia induction. In addition, although the remimazolam induction and PSI-guided titration strategy was protocolized, there is limited published evidence specifically defining the exact post-loss-of-consciousness maintenance adjustment range of 0.6–1.0 mg/kg/h in patients with severe aortic stenosis. In this study, we selected this range a priori based on our previous TAVR anesthesia protocol [9]; however, external validation of this specific dosing range in severe aortic stenosis is warranted. Third, anesthesia induction was performed without opioids, although co-administration of opioids could have influenced hemodynamic responses and HRV. Fourth, baseline HRV and hemodynamic measurements were obtained immediately before induction, when preoperative anxiety may have shifted autonomic balance toward sympathetic predominance [50]. This factor may have influenced baseline values. Fifth, the attending anesthesiologists were not blinded to HFVI during anesthesia induction. Awareness of HFVI values—particularly at the 2nd HFVI measurement—may have inadvertently influenced anesthetic management, constituting a potential source of bias. However, the attending anesthesiologists were unlikely to intentionally manipulate the HFVI values regardless of HFVI visualization, as the anesthetic protocols for both remimazolam and propofol were prespecified and rigorously implemented. We, therefore, consider that strict adherence to the standardized anesthetic protocols substantially minimized this potential bias. Finally, the second HFVI measurement was obtained during mask ventilation after rocuronium administration and before remifentanil infusion. Although this opioid-free window was intentionally chosen to minimize opioid-related confounding on HRV-derived indices (as similarly performed in prior induction HRV studies [10]), airway management maneuvers during mask ventilation may have acted as noxious stimuli and influenced HFVI and heart rate. Therefore, the observed between-group differences should be interpreted as differential autonomic patterns associated with the induction regimens under standardized conditions rather than definitive evidence of a direct causal drug effect on autonomic modulation.

In conclusion, this study compared the effects of remimazolam and propofol on parasympathetic activity, as assessed by HRV analysis, during anesthesia induction in patients with severe aortic stenosis. Remimazolam induction was associated with lower parasympathetic activity compared with propofol induction.

## Data Availability

The datasets supporting the findings of this study are available from the corresponding author upon reasonable request.
